# Degradation of Swainsonine by the NADP-Dependent Alcohol Dehydrogenase A1R6C3 in *Arthrobacter* sp. HW08

**DOI:** 10.3390/toxins8050145

**Published:** 2016-05-16

**Authors:** Yan Wang, A’guan Zhai, Yanqi Zhang, Kai Qiu, Jianhua Wang, Qinfan Li

**Affiliations:** 1 College of Veterinary Medicine, Northwest A & F University, No. 22 Xinong Road, Yangling 712100, China; zhaiaguan@163.com (A.Z.); zyq411069648@163.com (Y.Z.); jhwang1948@sina.com (J.W.); 2 Hulun Buir Animal Epidemic Prevention and Control Center, Hulun Buir 021000, China; jerry820312@126.com

**Keywords:** swainsonine, *Arthrobacter* sp., alcohol dehydrogenase, proteomics, degradation

## Abstract

Swainsonine is an indolizidine alkaloid that has been found in locoweeds and some fungi. Our previous study demonstrated that *Arthrobacter* sp. HW08 or its crude enzyme extract could degrade swainsonie efficiently. However, the mechanism of swainsonine degradation in bacteria remains unclear. In this study, we used label-free quantitative proteomics method based on liquid chromatography-electrospray ionization-tandem mass spectrometry to dissect the mechanism of swainsonine biodegradation by *Arthrobacter* sp. HW08. The results showed that 129 differentially expressed proteins were relevant to swainsonine degradation. These differentially expressed proteins were mostly related to the biological process of metabolism and the molecular function of catalytic activity. Among the 129 differentially expressed proteins, putative sugar phosphate isomerase/epimerase A1R5X7, Acetyl-CoA acetyltransferase A0JZ95, and nicotinamide adenine dinucleotide phosphate (NADP)-dependent alcohol dehydrogenase A1R6C3 were found to contribute to the swainsonine degradation. Notably, NADP-dependent alcohol dehyrodgenase A1R6C3 appeared to play a major role in degrading swainsonine, but not as much as *Arthrobacter* sp. HW08 did. Collectively, our findings here provide insights to understand the mechanism of swainsonine degradation in bacteria.

## 1. Introduction

Locoweed poisoning is a great threat to grass farming in the livestock industry [1]. Many lines of evidence have demonstrated that swainsonine (SW) is the main toxin in locoweeds [2,3,4]. Pathology studies showed that SW can inhibit cellular alpha-mannosidase and induce lysosomal accumulation of incompletely processed oligosaccharides as vacuoles in many cell types, including liver, renal, and cerebellar cells [5,6]. The pathological changes caused by SW directly or indirectly lead to the clinical symptoms of locoism, including birth defects, reproductive disorders, congestive heart failure, edema, growth retardation, and body weight loss [7,8]. Several methods have been used so far to prevent SW poisoning in livestock by either managing locoweeds or administering vaccines [9,10,11].

In our previous study, we isolated and characterized *Arthrobacter* sp. HW08 (hereafter strain HW08) as a potential SW-degrading bacterium [12]. A cell-free extract of strain HW08 could also effectively degrade SW *in vitro* [5]. However, the mechanism of SW biodegradation by strain HW08 is largely unknown. In this study, a label-free quantitative (LFQ) proteomics method using liquid chromatography-tandem mass spectrometry (LC-ESI-MS/MS) was used to identify the proteins in strain HW08 that were differentially expressed with or without SW stimulation. Using this assay, we demonstrated that the NADP-dependent alcohol dehydrogenase AAur_2040 played a dominant role in SW degradation.

## 2. Results

### 2.1. Degradation of SW by Strain HW08

**Figure 1:**
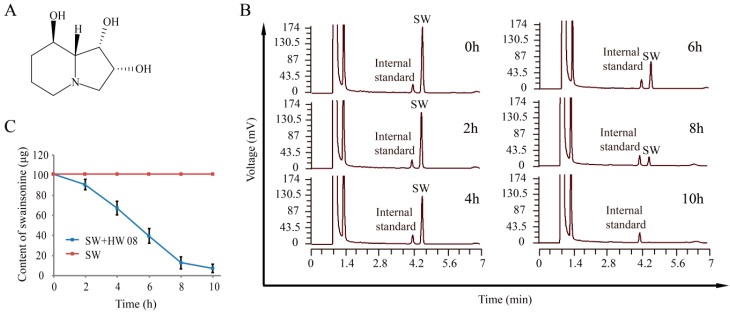
Determination of an optimal time point for analyzing swainsonine (SW)-degrading proteins in *Arthrobacter* strain HW08. (**A**) Chemical structure of SW; (**B**) Dynamics of SW degradation monitored by gas chromatography; (**C**) Quantification of SW degradation from 0 to 10 h.

The strain HW08 was previously isolated and was shown to have the capacity to degrade SW, namely (1S, 2R, 8R, 8aR)-1,2,3,5,6,7,8,8a-Octahydroindolizine-1,2,8-triol (Figure 1A). To determine the optimal time point for analyzing the SW-degrading proteins of strain HW08, GC analysis was performed to investigate the dynamic changes in SW degradation (Figure 1B). The results showed that SW degradation began within the first two hours of culture. Then, the SW content decreased linearly from 2 h to 8 h, and 900 micrograms of SW could be completely degraded within 10 h (Figure 1B,C). These results indicate that the levels of the SW-degrading enzymes of strain HW08 are likely highest 2–8 h after SW degradation began. Therefore, we chose 6 h after the start of SW degradation as the time point to harvest strain HW08 for the proteomics analysis.

### 2.2. LC-ESI-MS/MS Analysis of Strain HW08

**Figure 2:**
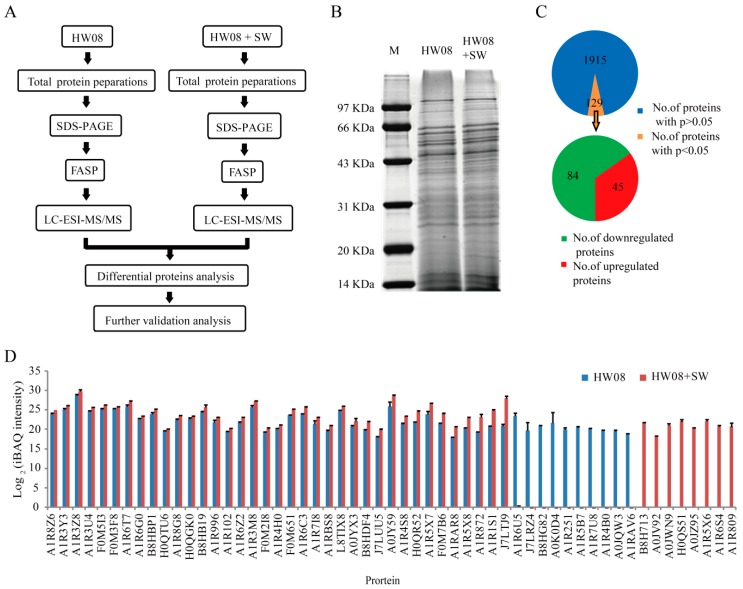
LC-ESI-MS/MS analysis of *Arthrobacter* strain HW08 cultured with SW. (**A**) Flow diagram of the LC-ESI-MS/MS analysis. FASP: Filter-aided sample preparation; (**B**) SDS-PAGE of total protein from strain HW08 cells cultured with and without SW; (**C**) Categorization of the 2044 proteins identified by LC-ESI-MS/MS; (**D**) Quantification of the 45 proteins that were upregulated in strain HW08 cells cultured with SW based on iBAQ intensity. Quantification of the 10 proteins that specifically expressed in strain HW08 without SW induction is also shown. Values are the mean of log2 (iBAQ intensity); error bars indicate standard derivation.

The LC-ESI-MS/MS analysis procedure is illustrated in Figure 2A. The strain HW08 cells cultured with and without SW (300 µg/mL) for 6 h were collected for protein analysis. After lysis and protein quantification using bicinchoninic acid (BCA), the proteins were separated by SDS-PAGE. The two samples, namely strain HW08 and strain HW08 + SW showed comparable total protein levels (Figure 2B). The A280 of strain HW08 and strain HW08 + SW were 1.2 and 1.4, respectively. The peptides were then analyzed by LC-ESI-MS/MS, and a total of 2044 proteins were identified. iBAQ analysis identified 129 differentially expressed proteins that were significantly relevant to SW degradation (*p* < 0.05) (Table S1). Among the 129 relevant proteins, 45 were found to upregulate their expression after SW induction. In contrast, 84 were found to downregulate their expression after SW induction (Figure 2C). The expression levels of the 45 upregulated proteins are shown in Figure 2D. Of note, eight proteins were found to be specifically expressed in strain HW08 with SW induction. Interestingly, 10 proteins were found to be expressed only in strain HW08 without SW induction (Figure 2D).

### 2.3. Bioinformatics Analysis of SW Degradation-Relevant Differentially Expressed Proteins

**Figure 3:**
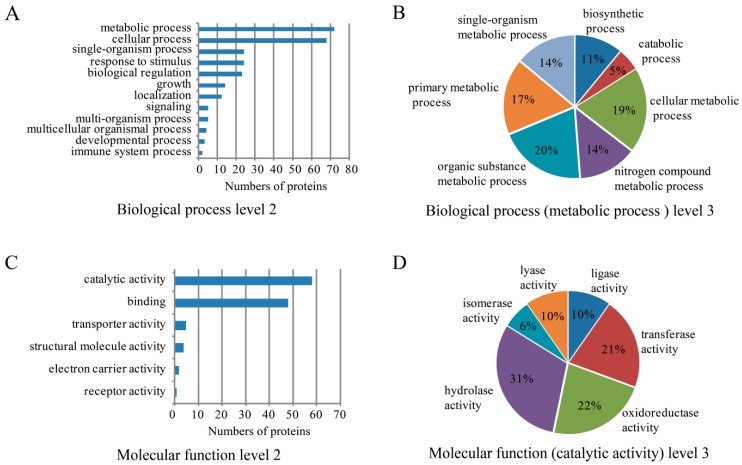
Bioinformatics analysis of differentially expressed proteins. (**A**) Gene ontology (GO) analysis of 129 differentially expressed proteins categorized by biological processes at level 2; (**B**) GO analysis of differentially expressed proteins enriched in metabolic process at level 3; (**C**) GO analysis of 129 differentially expressed proteins categorized by molecular function at level 2; (**D**) GO analysis of differentially expressed proteins enriched in catalytic activity at level 3. GO levels are defined by annotators to assign properties to gene products, which consist of three aspects of annotations at level 1, namely biological process, molecular function, and cellular component. The further annotations of items in level 1 are defined as level 2 and the more specific annotations of items in level 2 are defined as level 3.

Gene ontology (GO) analysis of the 129 differentially expressed proteins showed that metabolic processes, cellular processes, single-organism processes, and response to stimulus were the top four biological processes that were relevant to SW degradation (Figure 3A). We focused on metabolic processes since most of the differentially expressed proteins were enriched in this process (Figure 3B). The percentages of the differentially expressed proteins involved in organic substance metabolic process, cellular metabolic process, primary metabolic process, nitrogen compound metabolic process, single-organism metabolic process, biosynthetic process, and catabolic process were 20%, 19%, 17%, 14%, 14%, 11%, and 5%, respectively (Figure 3B). GO analysis of the 129 differentially expressed proteins based on molecular function indicated that most of the enriched proteins mainly functioned in catalytic activity and binding (Figure 3C). Further analysis of the catalytic activities showed that hydrolase activity, oxidoreductase activity, and transferase activity were the top three molecular functions (Figure 3D). Other catalytic activities, namely lyase activity, ligase activity, and isomerase activity, represented 10%, 10%, and 6%, respectively, of the differentially expressed proteins (Figure 3D).

Kyoto Encyclopedia of Genes and Genomes (KEGG) pathway analysis of the 129 differentially expressed proteins showed that they were enriched in 36 pathways (Table 1). Notably, four proteins, A0JWN9, A1R5W7, H0QR52, and A1R4F9, were enriched in purine metabolism. There were three proteins each in glycolysis/gluconeogenesis and one carbon pool by folate. The proteins involved in glycolysis/gluconeogenesis pathway were A1R6C3, H0QM48, and H0QSJ6. The proteins involved in the pathway of one carbon pool by folate were H0QHI2, A1R5R2, and A1R8Y7 (Table 1, Table S2).

**Figure 4:**
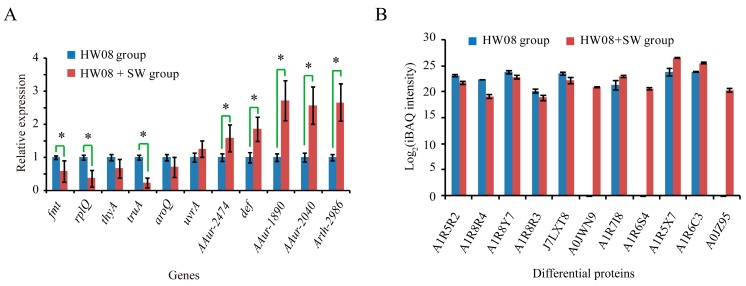
Validation of SW-degradation relevant differentially expressed proteins. (**A**) Real-time RT-PCR analysis of mRNAs encoding 11 differentially expressed proteins that participate in at least three biological processes and have catalytic activity; (**B**) Protein expression levels of the 11 differential genes based on iBAQ intensity. * indicates a *p* value < 0.05.

To further explore the molecular mechanism of the SW degradation pathway, we conducted a real-time RT-PCR validation analysis of 11 genes encoding proteins that participate in at least three biological processes and have catalytic activity from GO analysis. Among the 11 genes examined, the transcription levels of six genes were upregulated in the strain HW08 + SW cells compared to the levels in strain HW08 cells (Figure 4A). These real-time RT-PCR results were consistent with the protein quantification results based on iBAQ intensity (Figure 4B). Of note, *AAur_1890*, *AAur_2040*, and *Arth_2986*, which encode A1R5X7, A1R6C3, and A0JZ95, were the most significantly upregulated genes in strain HW08 cells following SW induction (Figure 4A,B). Then, we focused on the SW-degrading activity of these three candidates.

### 2.4. Screening and Validation of SW-Degrading Genes

**Figure 5:**
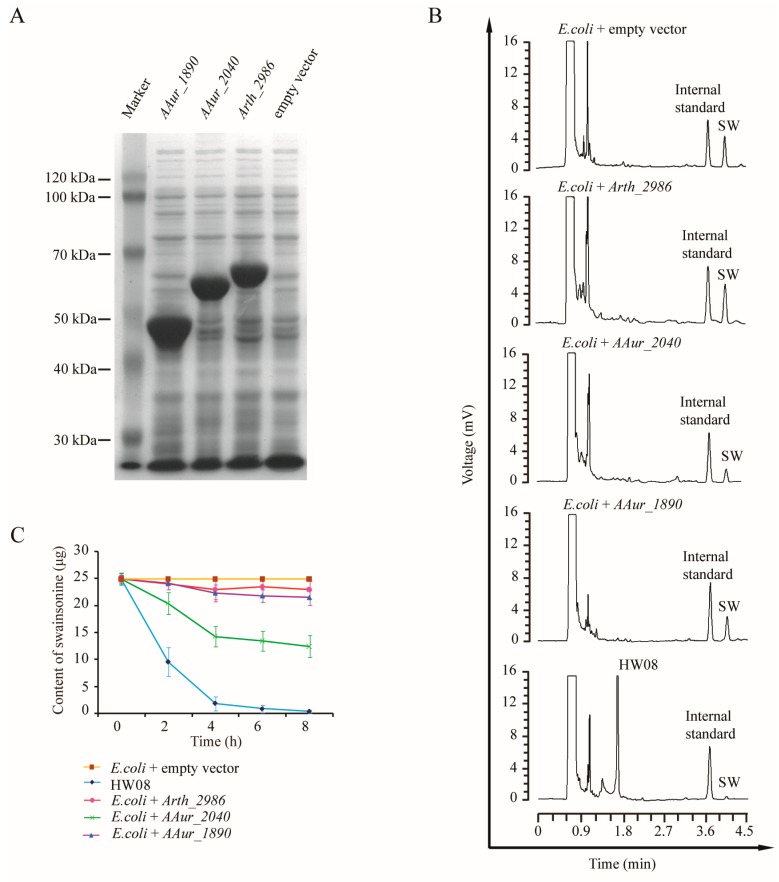
Screening and expression of critical SW-degradation genes. (**A**) SDS-PAGE of induced proteins; (**B**) GC analysis of SW degradation by *E. coli* BL21 (DE3) cells transformed with pET32α-*AAur_2040*, pET32α-*AAur_1890*, and pET32α-*Arth_2986*; (**C**) Time course of monitoring SW content in supernatant among different experimental groups. *E. coli* BL21 (DE3) cells transformed with pET32α-*AAur_2040*, pET32α-*AAur_1890*, and pET32α-*Arth_2986* were compared with strain HW08 and *E. coli* BL21 (DE3) cells control. Values are the mean from triplicates of independent experiments at each time point; error bars indicate standard deviation.

*AAur_1890*, *Arth_2986*, and *AAur_2040* were cloned into pET32a and confirmed by Sanger sequencing. When the three constructs containing *AAur_1890*, *Arth_2986*, and *AAur_2040* were transformed into *E. coli* BL21(DE3) cells, proteins of ~50, 60, and 65 kDa, respectively, were produced (Figure 5A). To investigate the SW-degrading capacity of the proteins encoded by *AAur_1890*, *Arth_2986*, and *AAur_2040*, *E. coli* BL21 (DE3) cells transformed with expression constructs containing these three genes were induced and were used in a SW-degrading assay. GC analysis confirmed the SW-degrading capacity of A1R5X7, A0JZ95, and A1R6C3 (Figure 5B). A time course of monitoring the SW content was performed (Figure 5C). Of note, the SW-degrading ratio of A1R6C3 reached up to 50.1% (12.53 μg/25 μg), and the ratios of A1R5X7 and A0JZ95 were 13.9% (3.48 μg/25 μg) and 8.2% (2.05 μg/25 μg), respectively (Figure 5C).

## 3. Discussion

In this study, LC-ESI/MS/MS analysis was used to identify SW-degrading genes of *Arthrobacter* strain HW08. Among the identified differentially expressed proteins, the NADP-dependent alcohol dehydrogenase A1R6C3 was shown to be the main candidate responsible for SW-degrading activity. These findings provide novel insights into the mechanism of SW degradation by *Arthrobacter*.

Interestingly, the metabolism of SW in eukaryotic cells is different from that in bacteria. SW can disrupt the metabolism of mannose and other glycoproteins *in vivo* [13,14], resulting in disorders of hormone and enzyme synthesis and receptor binding [15].

By contrast, some bacteria, like strain HW08, can degrade SW [12]. This property of strain HW08 is consistent with previous studies which showed that soil *Arthrobacter* can survive in stressful conditions induced by starvation, ionizing radiation, oxygen radicals, and toxic chemicals [16,17,18]. *Arthrobacter* can biodegrade a variety of environmental pollutants such as glyphosate [19], methyl tert-butyl ether [20], 2,4-dichlorophenoxyacetic acid [21], nicotine [22], 2-chloro-4-nitrophenol [23], dimethylsilanediol [24], endoxohexahydrophthalate [17], fluorine [25], phthalate [26], nitroglycerine [27], and a very large number of s-triazine herbicides [28]. The molecular bases for the degrading activity against these pollutants have been extensively investigated. In this study, LC-ESI/MS/MS analysis was used to dissect the mechanism of SW degradation by strain HW08. LC-ESI-MS/MS has several advantages, including its speed and simplicity, as well as the lack of a need for purification and derivatization. LC-ESI-MS/MS can also produce good results in terms of detection limits, repeatability, and linearity [29].

Most differentially expressed proteins in strain HW08 + SW belonged to dehydrogenases, including dehydrogenase (NADP+), dehydrogenase (acetyl-transferring), cinnamyl-alcohol dehydrogenase, dihydrolipoamide dehydrogenase (Table S2). NADP-dependent alcohol dehydrogenase A1R6C3, which is encoded by AAur_2040, was shown to degrade SW efficiently. Half concentration of SW could be nearly metabolized within eight hours. Co-transformation of the three candidate genes (*AAur_1890*, *Arth_2986*, and *AAur_2040*) into *Escherichia coli* was once tried in this study, but no significant improvement on SW-degradation was observed (Figure S1). Alcohol dehydrogenases mainly convert alcohols into their corresponding aldehydes or ketones. Among the three hydroxyl groups in SW chemical structure, the 1,2-di-hydroxyl is relatively unstable and may be oxidized by alcohol dehydrogenases with priority [30]. However, the process of biochemical reaction needs to be investigated in the future. Studies of NADP-dependent alcohol dehydrogenases appeared as early as in 1950s [31]. There are three classes of NADP-dependent ADHs, namely Zn-containing long-chain ADHs, short-chain metal-free ADHs, and Fe-containing/activated long-chain ADHs [32]. The protein encoded by AAur_2040 that was detected in strain HW08 + SW is a Zn-containing long-chain ADH according to the annotation of UniProt database. ADHs found in thermophilic and hyperthermophilic archaea and bacteria have diverse physiological roles [33]. KEGG analysis revealed that A1R6C3 participates in five pathways, including caprolactam degradation, glutathione metabolism, glycerolipid metabolism, glycolysis/gluconeogenesis, and phenylpropanoid biosynthesis (Table S2). A1R6C3 could nearly degrade a half concentration of SW within eight hours, which also suggests that A1R6C3 may be a critical target to understand the SW degradation in bacteria. Although exogenous NADP was not added in our SW-degradation testing culture medium, the endogenous NADP from transformed *Escherichia coli* may support the function of A1R6C3. Further studies on mechanisms of degrading SW by A1R6C3 and identification of the metabolite of SW are needed in the future.

## 4. Conclusions

In this study, putative sugar phosphate isomerase/epimerase A1R5X7, Acetyl-CoA acetyltransferase A0JZ95, and NADP-dependent alcohol dehydrogenase A1R6C3 were found to be candidate proteins responsible for SW-degradation in *Arthrobacter* strain HW08. Among the three candidates, A1R6C3 was identified to play a major role in degrading SW.

## 5. Materials and Methods

### 5.1. Bacterial Culture and Gas Chromatographic Analysis

The strain HW08 was deposited in China General Microbiological Culture Collection Center (CGMCC) with accession number 3313. SW was extracted from locoweed *Oxytropis glabra* (Lam.) DC. as described in our previous study [34]. SW above 97% purity was used in this study. In a SW degradation experiment, strain HW08 or *Escherichia coli* BL21 (DE3) cells transformed with single SW-degradation gene (*Arth_2040*, *Arth_1890*, or *Arth_2986*) were firstly grown in Luria-Bertani (LB) [35] broth for 24 h under the conditions described in our previous study [5]. Then the bacteria were harvested by centrifugation at 3000× *g* for 10 min and transferred into mineral salts medium (MSM) [36] with or without SW concentration of 300 µg/mL. The OD600 of bacteria was adjusted to 0.2 and cultured in a thermostatic shaker (200 rpm, 30 °C) for another 10 h. The bacteria samples (0.5 mL) were harvested every two hours during this period. The supernatant was collected by centrifuging (8000× *g*, 10 min) and lyophilized for gas chromatographic (GC) analysis.

GC analysis of SW was carried out as described previously with minor modifications [36]. Briefly, lyophilized supernatant samples were respectively dissolved in 30 μL solvent of pyridine and mixed with 50 μL internal standard of methyl α-d-galactopyranoside (me-Gal) (0.5 mg/mL) and 40 μL derivatization reagent of *N*,*O*-bis(trimethylsilyl)trifluoroacetamide with 1% trimethylchlorosilane. An aliquot of the mixture (2 μL) was injected for GC analysis. Shimadzu model 14C gas chromatograph equipped with a flame ionization detector (FID) and AT.SE-54 column were used in this study. The temperature of the column, injector port, and detector block were 210, 280, and 300 °C, respectively. Purified dry nitrogen was used as the carrier gas at a flow rate of 2 mL/min, and the split ratio was 60:1. The content of SW was determined by GC internal standard method [37].

### 5.2. LC-ESI-MS/MS Analysis

The pellet of strain HW08 bacteria with or without SW stimulation in MSM were suspended in 200 μL of lysis buffer (4% SDS, 100 mM DTT, 150 mM Tris-HCl pH 8.0) on ice, boiled, and then further lysed by sonication. The supernatant was collected, and total proteins were quantified with the BCA Protein Assay Kit (Bio-Rad, Hercules, CA, USA). After quantification, 20 µg of protein was separated by sodium dodecyl sulfate polyacrylamide gel electrophoresis (SDS-PAGE). Filter-aided sample preparation (FASP) was used to convert the proteins into peptides as described previously [38], and 5 µg of this peptide preparation was used for the LC-ESI-MS/MS analysis with a C18-reversed phase column (10 cm, 75 μm i.d., packed with 3 μm resin (Thermo, Waltham, MA, USA)) with triplicate samples. The mobile phases (A and B) were 2% ACN/0.1% formic acid and 84% ACN/0.1% formic acid, respectively. The flow rate was 400 nL/min. A 120-min linear gradient elution was used, which consisted of three periods, 0–100 min (0%–45% B), 100–108 min (45%–100% B), and 108–120 min (100% B).

The mass spectrometry (MS) experiments were performed on a Q-Exactive mass spectrometer coupled to an Easy-nLC (Proxeon Biosystems, now Thermo Fisher Scientific, Waltham, MA, USA). The spray voltage was 3.2 kV and the heated capillary was 300 °C with analysis time of 120 min. The scanning range was 300–1800 *m*/*z* and survey scans were acquired at a resolution of 70,000 at *m*/*z* 200. For MS/MS, the resolution was set to 17,500 at *m*/*z* 200. The detection mode was set to positive ion.

Label-free quantification was performed using MaxQuant (Max-Planck-Institute of Biochemistry, Am Klopferspitz, Germany) as previously described [39]. Six LC-ESI-MS/MS raw intensity files were used for intensity-based absolute quantification (iBAQ) [40]. Briefly, iBAQ values are the raw intensities divided by the number of theoretical peptides. In that way, iBAQ values are proportional to the molar quantities of the proteins. The MS data were searched against the UniProtKB *Arthrobacter* database, and the MaxQuant output files were subsequently uploaded into Perseus (Version 1.3.0.4, 2012, Max-Planck-Institute of Biochemistry, Am Klopferspitz, Germany) to calculate significance.

### 5.3. Gene Ontology and Kyoto Encyclopedia of Genes and Genomes Analysis

Gene ontology (GO) functional annotation of the differentially expressed proteins was performed using DAVID 2008 [41]. For the Kyoto Encyclopedia of Genes and Genomes (KEGG) analysis, the differentially expressed proteins were mapped to metabolic pathways in KEGG [42].

### 5.4. Gene Cloning and Expression

Among the differentially expressed genes in HW08 + SW, the upregulated genes which were mapped to more than four metabolic processes were selected as candidates for gene cloning. Genomic DNA from strain HW08 was isolated using a DNA Extraction Kit (TIANGEN, Beijing, China) according to the manufacturer’s instructions. Three candidate genes, *AAur_2040* (encoding protein A1R6C3), *AAur_1890* (encoding protein A1R5X7), and *Arth_2986* (encoding protein A0JZ95), were picked out of the differentially expressed genes and cloned into pET32a. The Genbank accession numbers of *AAur_2040*, *AAur_1890*, and *Arth_2986* are NC_008711 (region: 2241160...2242203), NC_008711 (region: 2066462...2067313), and NC_008541 (region: 3354284...3355483), respectively. The cloning primers are listed in Table S3. The sequence of the constructs was validated by Sanger sequencing (Sangon Biotech, Shanghai, China). For heterologous expression of A1R6C3, A1R5X7 and A0JZ95, *Escherichia coli* BL21 (DE3) cells (Beijing TransGen Biotech Co. Ltd., Beijing, China) were transformed with pET32α-*Arth_2040*, pET32α-*Arth_1890* and pET32α-*Arth_2986*, respectively. The transformed bacteria were cultured in LB medium containing 50 μg/mL ampicillin at 26 °C and 220 rpm. When the OD600 of culture reached 0.5, isopropyl β-d-thiogalactoside (IPTG) (Chemsynlab Phamaceutical Science & Technology Co. Ltd., Beijing, China) was added to the medium at a final concentration of 0.4 mmol/L. To induce the over-expression of these proteins, the culture was incubated overnight. Then, the bacteria were collected, lysed by ultrasonication, and ultracentrifuged at 12,000× *g* for 10 min. The supernatant containing the induced proteins was collected for SDS-PAGE analysis.

### 5.5. Real Time RT-PCR Analysis

Total RNA was isolated from ~10^8^ bacteria using the TRIzol kit (Invitrogen, Carlsbad, CA, USA) according to the manufacturer’s instructions. Total RNA was reverse transcribed to cDNA using the Reverse Transcriptase kit (Takara, Kusatsu, Japan). The primers used for real-time RT-PCR are listed in Table S4. Real-time RT-PCR amplification was carried out using the SYBR Premix Ex Taq II (Tli RNaseH Plus) Kit (Takara, Kusatsu, Japan) and the IQ5 Real-time PCR Detection System (170-9780, Bio-Rad, Hercules, CA, USA). The reaction conditions were as follows: initial denaturation at 95 °C for 30 s; 40 cycles of amplification with denaturation at 95 °C for 5 s and annealing and extension at 60 °C for 30 s; and one cycle of melting curve analysis at 95 °C for 5 s and 55 °C for 30 s. The real-time RT-PCR results were analyzed using 2^−ΔΔ*C*^^T^ method [43].

### 5.6. Statistical Analysis

The significance of the differences among different treatment groups was determined using a paired *t*-test. The level of significance was set at a *p*-value of less than 0.05 or 0.01.

## Figures and Tables

**Table 1:** Kyoto Encyclopedia of Genes and Genomes (KEGG) pathways analysis of 129 differentially expressed proteins.

Pathway	Number of Matched Pathways	Pathway	Number of Matched Pathways
Biotin metabolism	1	Phenylpropanoid biosynthesis	1
Pyruvate metabolism	2	Aminoacyl-tRNA biosynthesis	2
Glycolysis/Gluconeogenesis	3	Butanoate metabolism	2
Arginine and proline metabolism	2	Benzoate degradation	1
Fatty acid degradation	1	Purine metabolism	4
Glycine, serine and threonine metabolism	2	Pantothenate and CoA biosynthesis	1
Ubiquinone and other terpenoid-quinone biosynthesis	1	Glutathione metabolism	1
Styrene degradation	1	Caprolactam degradation	1
Ethylbenzene degradation	1	T cell receptor signaling pathway	1
Tyrosine metabolism	1	Fatty acid elongation	1
One carbon pool by folate	3	Fatty acid biosynthesis	2
Biosynthesis of unsaturated fatty acids	2	Alanine, aspartate and glutamate metabolism	1
Geraniol degradation	1	Valine, leucine, and isoleucine degradation	2
Thiamine metabolism	1	beta-Alanine metabolism	1
Nicotinate and nicotinamide metabolism	1	Glyoxylate and dicarboxylate metabolism	1
Citrate cycle (TCA cycle)	2	Glycerolipid metabolism	1
Pyrimidine metabolism	1	alpha-Linolenic acid metabolism	1
Phenylalanine, tyrosine and tryptophan biosynthesis	2	Carbon fixation pathways in prokaryotes	1

## Supplementary Materials

The following are available online at www.mdpi.com/2072-6651/8/5/145/s1, Table S1: List of 129 differentially expressed proteins; Table S2: List of GO terms and KEGG pathways; Table S3: Primers used for cloning Arth_2986, AAur_2040, and AAur_1890; Table S4: Primers used for real-time RT-PCR detection; Figure S1: SW degradation ratio of *E. coli* BL21 (DE3) cells when transformed with pET32α-*AAur_2040*, pET32α-*AAur_1890*, pET32α-*Arth_2986* or combination after 12 h.

## Abbreviations

The following abbreviations are used in this manuscript:

BCA	bicinchoninic acid
CGMCC	China General Microbiological Culture Collection Center
FASP	filter-aided sample preparation
FID	flame ionization detector
GC	gas chromatographic
GO	Gene ontology
iBAQ	intensity-based absolute quantification
IPTG	isopropyl β-d-thiogalactoside
KEGG	Kyoto Encyclopedia of Genes and Genomes
LB	Luria-Bertani
LC-ESI-MS/MS	liquid chromatography-tandem mass spectrometry
LFQ	label-free quantitative
me-Gal	methyl α-d-galactopyranoside
MS	mass spectrometry
MSM	mineral salts medium
SDS-PAGE	sodium dodecyl sulfate polyacrylamide gel electrophoresis
