# Supplementary material for: Degradation of Swainsonine by the NADP-Dependent Alcohol Dehydrogenase A1R6C3 in Arthrobacter sp. HW08
Source: Toxins (Basel). 2016 May 16;8(5):145. doi: 10.3390/toxins8050145 (PMC4885060; doi:10.3390/toxins8050145)
Supplement: Supplementary file 1 [file toxins-08-00145-s001.zip › toxins-124164-supplementary/Figure S1.pdf]

Supplementary Materials: Degradation of Swainsonine by the NADP-Dependent Alcohol Dehydrogenase A1R6C3 in *Arthrobacter* sp. HW08

Yan Wang, A'guan Zhai, Yanqi Zhang, Kai Qiu, Jianhua Wang, and Qinfan Li

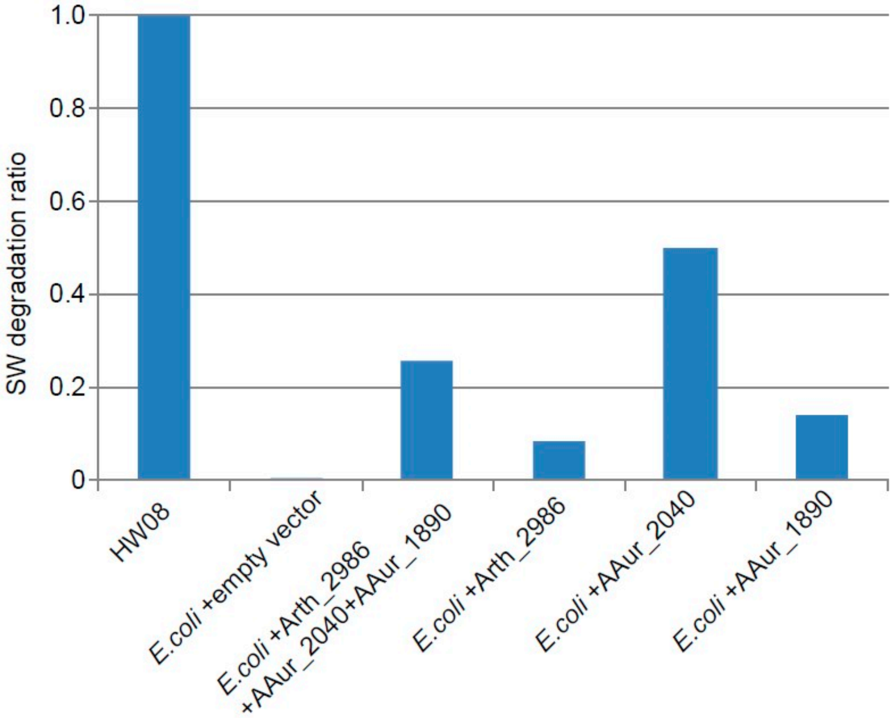

Figure S1. SW (Swainsonine) degradation ratio of *E. coli* BL21 (DE3) cells when transformed with pET32α-*AAur_2040*, pET32α-*AAur_1890*, pET32α-*Arth_2986* or combination after 12 h.
